# Comparative evaluation of disc diffusion and broth microdilution methods for aztreonam/avibactam susceptibility testing in Enterobacterales

**DOI:** 10.1093/jac/dkaf361

**Published:** 2025-09-26

**Authors:** Ioannis Baltas, Deny Tsakri, Sofia Vourli, Himani Solanki, Evelyn Murrell, Eftychia Kiousi, Zacharias Tsakris, Gabriella Kuczmar, Nikoletta Smyrni, Ioannis Skiadas, Vassilios Grammelis, James Hatcher, Damianos Menegas, Athanasios Tsakris, Georgia Vrioni, Louis Grandjean

**Affiliations:** Infection, Immunity & Inflammation Department, UCL Institute of Child Health, London, UK; Infection, Immunity & Inflammation Department, UCL Institute of Child Health, London, UK; Institute of Biosciences and Applications, NCSR ‘Demokritos’, Athens, Greece; Infection, Immunity & Inflammation Department, UCL Institute of Child Health, London, UK; Infection, Immunity & Inflammation Department, UCL Institute of Child Health, London, UK; Infection, Immunity & Inflammation Department, UCL Institute of Child Health, London, UK; Infection, Immunity & Inflammation Department, UCL Institute of Child Health, London, UK; Department of Clinical Microbiology, Great Ormond Street Hospital for Children, London, UK; Pfizer Hellas, Athens, Greece; Pfizer Hellas, Athens, Greece; Pfizer Hellas, Athens, Greece; Infection, Immunity & Inflammation Department, UCL Institute of Child Health, London, UK; Department of Clinical Microbiology, Great Ormond Street Hospital for Children, London, UK; Pfizer Hellas, Athens, Greece; Department of Microbiology, Medical School, National and Kapodistrian University of Athens, Athens, Greece; Department of Microbiology, Medical School, National and Kapodistrian University of Athens, Athens, Greece; Infection, Immunity & Inflammation Department, UCL Institute of Child Health, London, UK

## Abstract

**Background:**

Aztreonam/avibactam is a novel β-lactam/β-lactamase inhibitor (BL/BLI) combination active against carbapenem-resistant Enterobacterales (CRE), including MBL-producing isolates. In May 2024, EUCAST published Enterobacterales breakpoints for aztreonam/avibactam. This study aimed to assess the performance of commercially available disc diffusion (DD) against broth microdilution (BMD) using the latest EUCAST breakpoints.

**Methods:**

We tested 278 CRE causing infections in 17 Greek ICUs between 2021 and 2023, using 30/20 μg aztreonam/avibactam discs and BMD according to EUCAST methodology and EUCAST version 15.0 breakpoints.

**Results:**

Most isolates were identified as *Klebsiella pneumoniae* (97.8%), and 98.9% produced carbapenemases, including 46.4% KPC, 20.1% NDM, 5.4% VIM and 27% multiple carbapenemases. Using BMD, 94.2% of isolates were susceptible to aztreonam/avibactam. Conversely, using DD, 66.9% were susceptible, 33.1% resistant and 27% within the area of technical uncertainty (ATU). Most isolates in the ATU were KPC-producing (68%) or KPC and MBL-producing (29.3%). All isolates in the ATU (22–24 mm) tested susceptible by BMD (MIC ≤ 4 mg/L). One isolate exhibited resistance by DD (20 mm), but was susceptible by BMD. Categorical agreement (CA) was 72.7%, with 29% major errors (MEs) and 0% very major errors (VMEs). Using a breakpoint of 22 mm, CA, ME and VME were 99.6%, 0.4% and 0%, respectively.

**Conclusions:**

Aztreonam/avibactam showed potent *in vitro* activity against MBL- and KPC-producing Enterobacterales. Using the 2025 EUCAST breakpoint, all isolates in the ATU tested susceptible by BMD, leading to high MEs of the DD method. A 22 mm breakpoint would have corrected this discrepancy in our cohort.

## Introduction

There is a significant global health threat posed by carbapenem-resistant Enterobacterales (CRE), especially MBL-producing isolates, where treatment options are limited.^1^ Most novel β-lactam/β-lactamase inhibitor (BL/BLI) combinations lack activity against MBL-producing infections, and resistance to cefiderocol has been widely documented.^2,3^ Aztreonam/avibactam is a novel BL/BLI combination that demonstrates potent activity against MBL-producing CRE.^4^ This is because aztreonam is stable against MBL-mediated hydrolysis, whereas avibactam strongly inhibits any potential co-existing serine β-lactamases.^5,6^ Although resistance to aztreonam/avibactam among MBL-producers is rare, it has been described in the form of PBP-3 and porin mutations.^6^ Therefore, precise and prompt susceptibility testing of aztreonam/avibactam is crucial for the treatment of infections with MBL-producing CRE.

In May 2024, EUCAST introduced Enterobacterales MIC and zone diameter breakpoints for aztreonam/avibactam, whereas the CLSI has yet to set its own.^7^ Testing options remain limited to reference broth microdilution (BMD), disc diffusion (DD) and gradient diffusion; no automated antimicrobial susceptibility testing (AST) platforms currently support this combination.^8^ In October 2024, aztreonam/avibactam discs became commercially available for the first time. DD has potential advantages, due to its cost-effectiveness, rapid turnaround time and ease of use, yet correlation with the reference method (BMD) is recommended. In this study, we aimed to investigate the correlation of aztreonam/avibactam DD with BMD using the recently published 2025 EUCAST breakpoint for Enterobacterales.

## Materials and methods

### Bacterial isolates

A total of 278 non-duplicate CRE were consecutively collected from patients with documented CRE infections in 17 Greek ICUs between May 2021 and December 2023 as part of the prospective, non-interventional, nationwide INCREASE cohort study (Ethics Ref. No. C3591034), sponsored by Pfizer Hellas SA.^9^ Bacterial species were identified using MALDI-TOF MS with the Bruker Biotyper^®^ system (Bruker Daltonics GmbH & Co. KG, Bremen, Germany). Carbapenem resistance was confirmed using the MICRONAUT-S MDR MRGN screening panel (Bruker Daltonics GmbH & Co. KG, Bremen, Germany). Carbapenemases were detected using Carbaplex^®^ IVD (Bruker Daltonics GmbH & Co. KG, Bremen, Germany).

### Aztreonam/avibactam susceptibility testing

Aztreonam/avibactam BMD was performed using a custom-made dried BMD plate (GB1UCLNF) for the Thermo Scientific^™^ Sensititre^™^ ARIS HiQ^™^ System (Thermo Fisher Diagnostics Ltd, Basingstoke, UK). Aztreonam concentration ranged from 0.03 to 64 mg/L with a fixed avibactam concentration of 4 mg/L. Isolates were tested according to manufacturer’s instructions using the high inoculum method (30 μL).^10^ MICs were read manually using the Sensititre^™^ Vizion^™^ Digital MIC Viewing System (Thermo Fisher Diagnostics Ltd, Basingstoke, UK), calibrated to manual inverted mirror reading for this plate. DD was performed using MASTDISCS^®^ aztreonam 30 μg/avibactam 20 μg cartridge discs (AZA50C; Mast Group Ltd, Bootle, UK) according to the EUCAST DD method on pre-poured, pre-prepared unsupplemented Mueller–Hinton agar (Oxoid Limited, Basingstoke, UK). The diameter of the inhibition zone was measured using a calibrated ruler against a dark background.

For both methods, incubation was conducted at 35 ± 1°C for 16–20 h in ambient air. Purity checks were performed for each isolate before AST results were interpreted. Daily quality control (QC) was performed using *Klebsiella pneumoniae* ATCC 700603. For all isolates, susceptibility results were confirmed by two investigators, who were blinded to BMD results when interpreting DD results and vice versa.

### Results analysis

Aztreonam/avibactam susceptibility results were determined using EUCAST 2025 v15.0 breakpoints (MIC breakpoint ≤4 mg/L, zone diameter breakpoint ≥25 mm susceptible, 22–24 mm ATU, <25 mm resistant).^7^ Categorical agreement (CA), major error (ME) and very major error (VME) were computed in accordance with CLSI M52.^11^ In short, CA is achieved when both the test method (DD) and the reference method (BMD) assign the same interpretive category (Susceptible [S]; Susceptible, increased exposure [I]; or Resistant [R]) to a microbial isolate (target ≥90%); ME occurs when a susceptible isolate is incorrectly categorized as resistant by DD (target ≤3%); VME occurs when a resistant isolate is incorrectly categorized as susceptible by DD (target ≤1.5%).

## Results

### Profiling of clinical isolates

A total of 278 CRE were included in the study, isolated in blood (68.3%, 190/278), bronchoalveolar lavage (10.8%, 30/278), sputum (9.7%, 27/278), urine (5%, 14/278) and other sites (6.2%, 17/278). *K. pneumoniae* accounted for 97.8% (272/278) of all study isolates. All isolates were resistant to ertapenem, 98.9% (275/278) to imipenem and 90.3% (251/278) to meropenem. In total, 98.9% (275/278) of isolates produced carbapenemases, including 46.4% KPC (129/278), 20.1% NDM (56/278) and 5.4% (15/278) VIM. Double carbapenemase production was detected in 27% of isolates (75/278), including 15.8% (44/278) KPC+VIM, 10.1% (28/278) KPC+NDM, 0.7% (2/278) NDM+OXA-48, and 0.3% (1/278) NDM+ VIM+KPC.

### AST results

**Figure 1.**
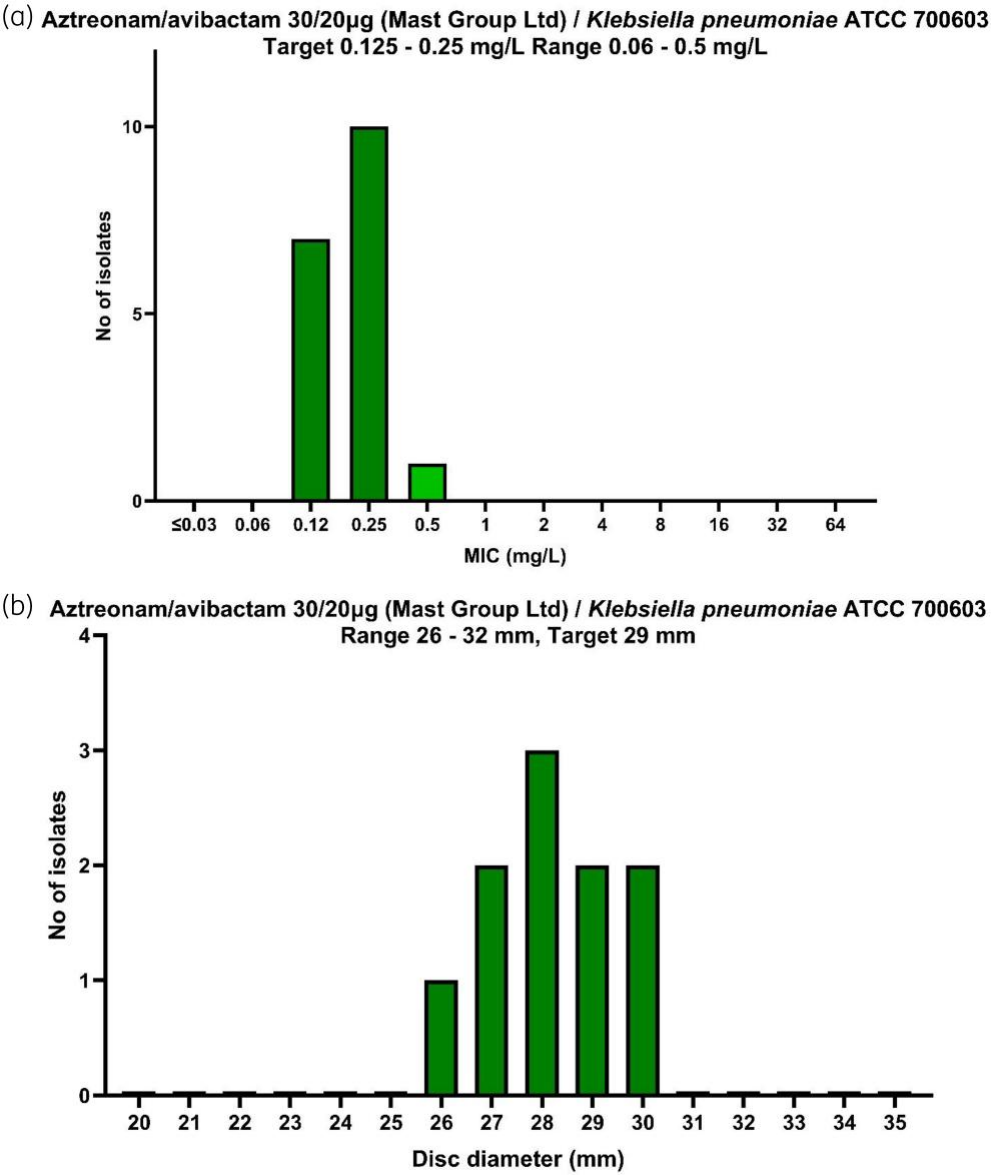
Results of QC isolate *K. pneumoniae* ATCC 700603 against aztreonam/avibactam (a) using BMD shown in mg/L and (b) DD method shown in mm. EUCAST range and target values are shown respectively. QC testing was performed at least once on each day of testing.

All QC results were within range (Figure 1). BMD QC results were on target 94.4% (17/18) of the time. The mode, median and average QC disc diameters were 28, 28.5 and 28.3 mm respectively, slightly lower than the 29 mm EUCAST QC target but within the accepted QC range of 26–32 mm.

### EUCAST

**Figure 2.**
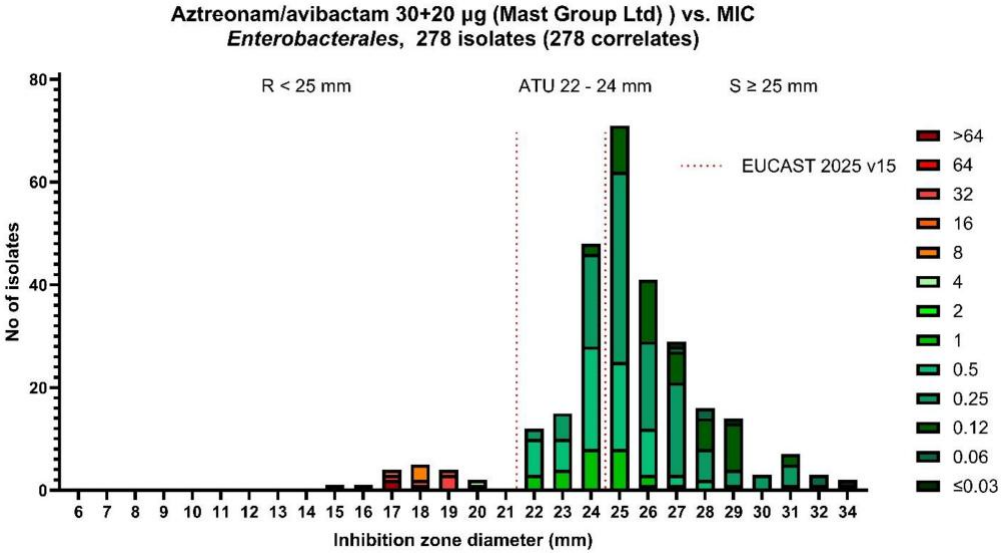
Aztreonam/avibactam inhibition zone diameter in correlation with the MIC values for all isolates (*N* = 278), according to EUCAST 2025 version 15.0 breakpoints. Susceptible (S), within the ATU (ATU), and resistant (R) values are shown.

BMD identified 94.2% (262/278) of Enterobacterales as susceptible; 5.8% (16/278) were resistant: 7 KPC+VIM, 5 KPC-only, 2 VIM-only, 1 NDM+KPC, and 1 NDM-only (15 were *K. pneumoniae* and 1 was *Proteus mirabilis*). The 16 resistant isolates were collected in 10 different ICUs. Using DD, 66.9% (186/278) of the isolates tested susceptible and 33.1% were resistant (92/278); 27% (75/278) were within the ATU. All isolates in the ATU were *K. pneumoniae*. The majority of isolates in the ATU were KPC-producing (51/75) or double carbapenemase-producing (9 KPC+NDM; 13 KPC+VIM), whereas only 2 were MBL-only–producing (NDM 1, VIM 1). Plotting DD versus BMD (Figure 2) showed clear separation: all ATU isolates (22–24 mm) were BMD-susceptible (MIC range 0.12–1 mg/L), except one isolate (20 mm DD, MIC 4 mg/L) that BMD still deemed susceptible. Consequently, using the 25 mm EUCAST v15.0 cutoff, CA was 72.7% (202/278), with 29% (76/262) ME and 0% (0/16) VME, mainly due to ATU ambiguity. Removing ATU isolates (*n* = 75) pushed CA to 99.5% (202/203) and ME down to 0.5%. Applying a 22 mm breakpoint yielded CA 99.6% (277/278), ME 0.4% (1/262), 0% (0/16) VME.

## Discussion

In this study, we evaluated the performance of the first commercially available aztreonam/avibactam DD test against BMD in a large multicentre collection of Greek ICU CRE isolates. Aztreonam/avibactam demonstrated strong *in vitro* activity against this cohort of XDR isolates, corroborating existing evidence of its potency, including against MBL-producers and double carbapenemase producers. These results support the most recent Greek Infectious Diseases Society Guidance on the management of MDR Gram-negative infections, where aztreonam/avibactam is recommended as first line for MBL-producing CRE.^12^ Some resistance was seen, primarily among *K. pneumoniae* isolates, in which the molecular underpinning of aztreonam/avibactam resistance is poorly understood, in contrast to *Escherichia coli*.^6^ Additionally, our results suggest that, in our collection of isolates, the current EUCAST DD aztreonam/avibactam breakpoint was predictive of susceptibility but not of resistance within the ATU. All ATU isolates tested susceptible using BMD, leading to high ME rates. This was particularly prominent for KPC and KPC+MBL-producing isolates. For this reason, isolates with disc diameters in the ATU should be tested using BMD, especially in patients with severe infections, as per current EUCAST guidance for isolates in the ATU.

To our knowledge, only one other study has previously evaluated the DD breakpoint of aztreonam/avibactam: Yin *et al*^13^ assessed in-house developed discs of 30/20 and 10/4 μg against in-house BMD using an MIC breakpoint of 8 mg/L and found high rates of VME, which were absent in our study. Given the use of in-house testing and an alternative MIC breakpoint, it is difficult to contextualize the results of this study within our work. EUCAST used a collection of 201 isolates for determining the aztreonam/avibactam breakpoint, where overlap of resistant and susceptible isolates between 21 and 24 mm was seen.^7^ On the contrary, a clear cutoff at 22 mm was observed in our study. Additional information on isolates, species and carbapenemase production is not available from the EUCAST publication, although it should be noted that a higher proportion of resistant isolates was present compared with our study.

Strengths of our study include the high number of clinically relevant CPE isolates, for which aztreonam/avibactam is likely to be used in real life, as well as the use of high-quality commercially available BMD and DD solutions, which should make our results reproducible. We performed and presented QC results as per EUCAST recommendations. Limitations included the predominance of *K. pneumoniae* in our collection, which might partly explain differences of our results with EUCAST. We cannot exclude clonality, but the INCREASE study deliberately enrolled only one eligible isolate per patient across 17 ICUs in Greece, thereby reducing the risk as much as practically possible. Additionally, we did not perform repeat testing of isolates to obtain multiple replicates due to resource limitations. We only used a single agar supplier and did not assess a different brand of discs (Aztreonam/Avibactam (30 + 20) µg Disc; Liofilchem, Abruzzi, Italy), as these became available after the end of our study. We used a 30 μL inoculum method for BMD, which does slightly deviate from the ISO-recommended 50 μL volume, yet is supported both by the manufacturer and the literature.^10^ Finally, our QC results for DD were 0.5–1 mm smaller than the target diameter on average, which may have led to slight underestimation of disc diameters and slight overestimation of ME. Given our isolates were from a single country only, larger multicentre validation studies are needed to verify our findings.

In conclusion, aztreonam/avibactam showed potent *in vitro* activity against MBL- and KPC-producing Enterobacterales, supporting its use as a viable therapeutic option for CRE infections. Using the 2025 EUCAST breakpoint, all isolates in the ATU tested susceptible by BMD, leading to high ME of the DD method. A provisional disc diffusion breakpoint of ≥22 mm would have corrected most observed VMEs thereby improving categorical agreement between DD and BMD methods.

## Transparency declarations

I.B. reports investigator-initiated research funding from Shionogi B.V. for research on Gram-negative infections outside the submitted work; honoraria from Shionogi B.V., Menarini UK and Tillotts Pharma; and participating in advisory boards for GSK and Eumedica. N.S., I.S., V.G. and D.M. are employees of Pfizer and may hold stock or stock options. J.H. reports honoraria from Gilead and Pfizer; participating in advisory boards for Gilead, Pfizer and Eumedica; and investigator-initiated research funding from Gilead. L.G. reports investigator-initiated research funding from Shionogi B.V., Pfizer and Advanz Pharma. All other authors report no conflicts of interest.
